# Wnt Signaling Induces Asymmetric Dynamics in the Actomyosin Cortex of the *C. elegans* Endomesodermal Precursor Cell

**DOI:** 10.3389/fcell.2021.702741

**Published:** 2021-09-17

**Authors:** Francesca Caroti, Wim Thiels, Michiel Vanslambrouck, Rob Jelier

**Affiliations:** Predictive Genetics and Multicellular Systems, CMPG, University of Leuven, Leuven, Belgium

**Keywords:** cell shape, asymmetric division, E-cadherin, Wnt signaling, *Caenorhabditis elegans*, nonmuscle myosin, F-actin (filamentous actin), cellular cortex

## Abstract

During asymmetrical division of the endomesodermal precursor cell EMS, a cortical flow arises, and the daughter cells, endodermal precursor E and mesodermal precursor MS, have an enduring difference in the levels of F-actin and non-muscular myosin. Ablation of the cell cortex suggests that these observed differences lead to differences in cortical tension. The higher F-actin and myosin levels in the MS daughter coincide with cell shape changes and relatively lower tension, indicating a soft, actively moving cell, whereas the lower signal in the E daughter cell is associated with higher tension and a more rigid, spherical shape. The cortical flow is under control of the Wnt signaling pathway. Perturbing the pathway removes the asymmetry arising during EMS division and induces subtle defects in the cellular movements at the eight-cell stage. The perturbed cellular movement appears to be associated with an asymmetric distribution of E-cadherin across the EMS cytokinesis groove. ABpl forms a lamellipodium which preferentially adheres to MS by the E-cadherin HMR-1. The HMR-1 asymmetry across the groove is complete just at the moment cytokinesis completes. Perturbing Wnt signaling equalizes the HMR-1 distribution across the lamellipodium. We conclude that Wnt signaling induces a cortical flow during EMS division, which results in a transition in the cortical contractile network for the daughter cells, as well as an asymmetric distribution of E-cadherin.

## Introduction

Understanding how cells self-organize during development into multicellular systems is a fundamental challenge in biology. One formative process during self-organization is the breaking of symmetry, particularly by polarization of cells and asymmetric divisions. A central role is played by the cellular cortex, a thin, highly dynamic actomyosin mesh just underneath the membrane of eukaryotic cells. Non-muscular myosin acts on the actin fibers, generates forces, and creates tension in the cortical network. Uneven tension in the cortex drives cell shape changes, for example during morphogenesis (Lecuit and Lenne, 2007). The cortex further contributes to shape changes during mitosis, and plays an important role in cell polarization and asymmetrical divisions.

The first asymmetric stem cell–like division of the *Caenorhabditis elegans* embryo has been extensively used as a model for polarized divisions. Directed contractility of the actomyosin cortex and the PAR system work together to establish the polarity of the zygote (Lang and Munro, 2017). Following a polarizing signal, in the form of the microtubule organizing center introduced by the sperm cell, an actomyosin cortical flow is initialized. This flow segregates a subset of PAR proteins into the nascent anterior, which facilitates the establishment of a posterior domain with a different set of PAR proteins (Dickinson et al., 2017; Lang and Munro, 2017; Wang et al., 2017). In other cells cortical flows were shown to have a surprising role in cellular positioning. Chiral counter rotating flows participate in the orientation of the mitotic spindle in several cells during early embryogenesis, which is important for left-right asymmetry breaking during early embryogenesis (Pimpale et al., 2020).

Here we aimed to identify further asymmetrical cortical processes by observing non-muscular myosin (NMY-2) and filamentous actin (F-actin) during the divisions of the early *C. elegans* embryo. We found a striking asymmetrical pattern in the EMS division at the four-cell stage. The EMS division is asymmetric, with the daughter E giving rise to endoderm and MS to muscle cells, among other cell types (Rose and Gönczy, 2014). The EMS cell is polarized along the A/P axis, not by the PAR system, but by a partially redundant Wnt and MES-1 signal from the neighboring P2 cell (Bei et al., 2002). In this paper, we characterize the cortical behavior of EMS and its descendants and study how it is affected by perturbation of the Wnt pathway.

## Materials and Methods

### *C. elegans* Strains and Maintenance

*Caenorhabditis elegans* strains were grown on NGM plates at 20˚C as previously described (Brenner, 1974). The strains used in this study are listed in Table 1.

**Table 1:** *C. elegans* strains used in this work.

**Strain**	**Genotype**	**Resources**
SWG001	gesls001[Pmex-5::Lifeact::mKate2::nmy-2UTR, unc-119+]	Reymann et al., 2016
LP306	cpIs53 [mex-5p::GFP-C1::PLC(delta)-PH::tbb-2 3'UTR + unc-119 (+)] II	Heppert et al., 2016
LP172	hmr-1(cp21[hmr-1::GFP + LoxP]) I	Marston et al., 2016
RW10029	zuIs178 [his-72(5' UTR)::his-72::SRPVAT::GFP::his-72 (3' UTR) + unc-119(+)].	Bao et al., 2006
stIs10024 [pie-1::H2B::GFP::pie-1 3' UTR + unc-119(+)]	
LP162	nmy-2(cp13[nmy-2::GFP + LoxP]) I.	Dickinson et al., 2013
RJ001	Cross between LP172 and SWG001	This work
RJ006	Cross between RW10029 and SWG001	This work
RJ012	Cross between LP162 and SWG001	This work
RJ013	Cross between LP306 and SWG001	This work

###  RNAi Experiments

RNAi was performed by feeding as described (Kamath et al., 2000). Briefly, L4 hermaphrodites were placed on NGM plates containing 25 μg/ml carbenicilline, 1 mM IPTG and seeded with bacteria expressing dsRNA. The induction was performed at 37°C for 4 h. The clones for *mom-2, dsh-2*, and *mig-5* RNAi were from the Vidal library. For all experiments a negative control (empty vector pL4440) was included to avoid scoring phenotypes not linked to the gene-specific RNAi. Phenotypes were scored after 48 h incubation at 20°C by *in vivo* imaging.

###  Microscopy

Long term imaging of the embryos was performed with a Zeiss LSM880 microscope using the fast-super resolution AiryScan mode and a Plan-Apochromat 63x/1.4 DIC M27 oil immersion objective. Z-stacks were acquired every 0.5 μm. The embryos were mounted on slides with M9 and Polybead Microspheres 20 μm (Polysciences) were used as spacer. The embryos were imaged every 2 min, except for the cell shape analysis (1.5 min) and cortical flows (5 s).

###  Ablation of the Cell Cortex

Ablations were performed with a 355 nm pulsed laser UGA-42 Caliburn from Rapp OptoElectronic mounted on a Zeiss LSM880. The cell cortex of a single cell was ablated in the anterior-posterior direction for 300 ms along a line of 4 μm at 11.3% laser strength. Ablations were performed after divisions were fully completed, several minutes after completion of cytokinesis. For E and MS this implied P2 division was at least underway, but more often P2 cytokinesis had already completed. Experiments were only included if the cell showed rapid healing of the cut, and proceeded to divide at least once. To detect the cytoskeleton dynamics, the time lapses were recorded as a single plane imaged at a rate of ±1 fps. The RJ012 strain was used for the ablation experiments, using the Airyscan detector in R/S mode for imaging the cortex and exciting NMY-2::GFP and the Lifeact peptide by 488 and 561 nm laser light, respectively. The two fluorophores were either excited together, or in separate frames.

###  Analysis of Cortical Mechanical Characteristics

The physical properties typically measured through a cortical ablation experiment are cortical tension and cortical stiffness. As laid out in Mayer et al. (2010), cortical tension is proportional to the velocity away from the ablation line immediately after the ablation (*v*_⊥, 0_), and the stiffness is inversely proportional to the relaxation time of the velocity (τ). The relaxation time is given by τ = ζ/*k*, where *k* is the elastic stiffness of the cortex and ζ characterizes frictional interactions between the cortex and the surrounding fluid. Because the fluid in which the cortex is embedded is likely to be relatively uniform throughout the embryo, differences in this parameter are expected to reflect differences in stiffness.

To quantify cortical velocities after ablation we used a custom-made Particle Image Velocimetry (PIV) analysis pipeline, based on manually annotated markers. These markers were placed on either the NMY-2 or F-actin channel using an in-house lineaging tool over a 7 s time frame (Supplementary Figures, Table 1). All markers were placed within 2.5 microns distance orthogonal to the cut and at every time interval within the time frame. Three ABpl cells displayed noticeable cortical flow, which was separately annotated using markers in close vicinity, but sufficiently distant from the cut as to not be affected by the ablation retraction. We subsequently corrected for flow by averaging the flow velocities per time step and subtracting the resulting mean flow velocity vector from the velocity field.

As in Mayer et al. (2010), the dynamics of the velocity component orthogonal to the cut line were modeled as a simple viscoelastic response, with the characteristic exponential decay over time: $v_{⊥}\left(t\right)=v_{⊥,0}e^{-t/\tau }$. Given markers were only tracked for 6 s post-cut, cortical resealing dynamics were not taken up in the model. A non-linear least squares method from the SciPy package was used to fit the model.

To obtain overall fits and confidence intervals fits for *v*_⊥, 0_ and τ a bootstrap was performed (*n* = 1,000) on the total data set of outward velocities, weighted to ensure sampling from each experiment with equal probability. The significance for contrasts between cells was also derived via a weighted bootstrap approach (*n* = 1,000) for every cell pair that was compared. A null distribution was simulated based on a sampling from the total set of outward velocities for that cell pair. Cell labels were randomly shuffled within this bootstrap dataset. The absolute value of the difference between the parameter inference for the compared cells was subsequently compared to the actual observed parameter difference for that cell pair. The *p*-value is then defined as the proportion of bootstraps that resulted in an equal or higher difference.

###  Image Analysis and Quantification

The confocal images were processed using the free software Fiji (https://imagej.net/Fiji). To quantify the Lifeact::MKATE-2 and NMY-2::GFP intensity at the cell cortex a maximum intensity projection was made for the z-planes containing the cell cortex specific signal. For every cell at each time point, a cell outline was drawn manually before measuring the cortical signal intensity.

###  Statistical Analyses and Visualization

**Figure 1:**
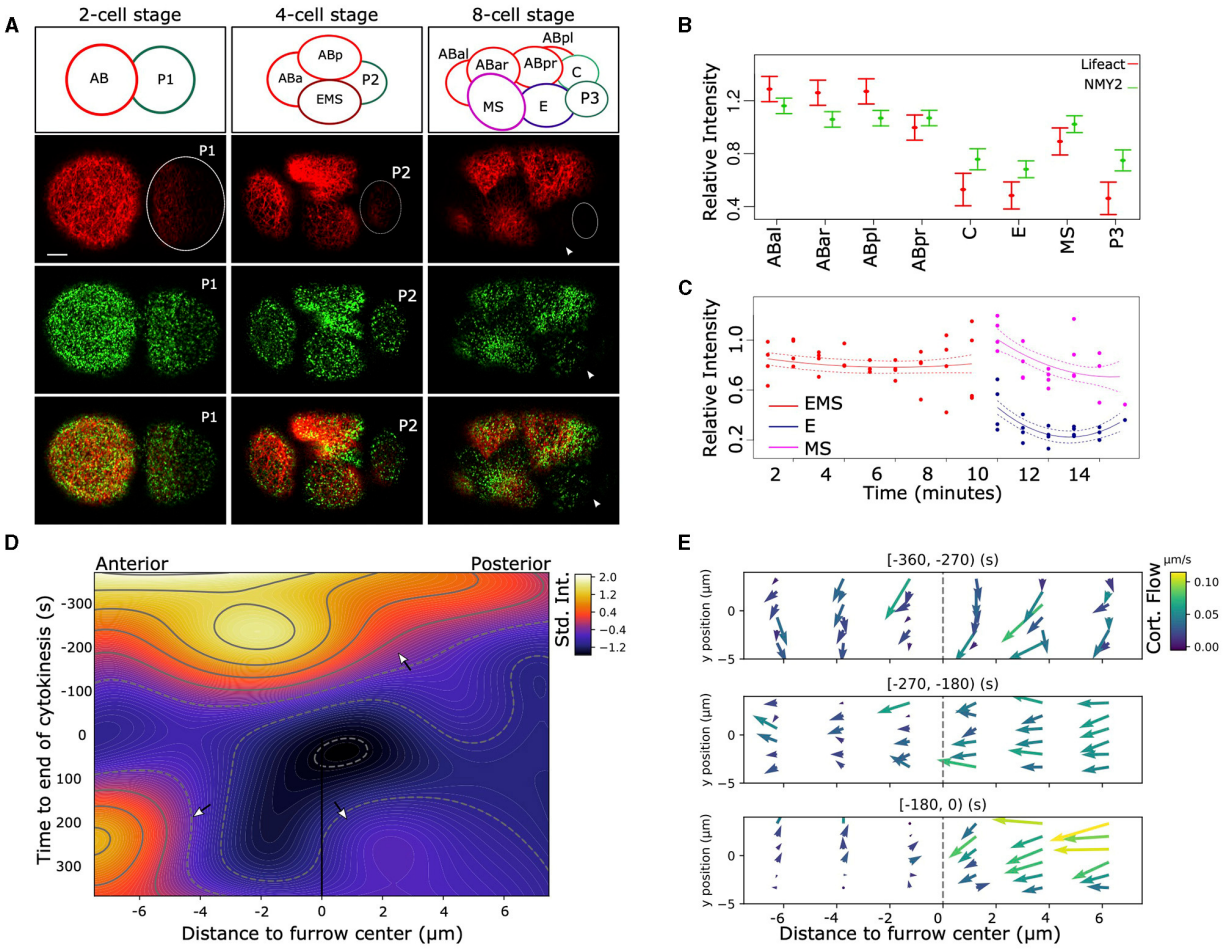
Asymmetric distribution of F-actin and NMY-2 in the early *C. elegans* embryo. **(A)** Asymmetrical distribution in the amount of F-actin (Lifeact, red) and non-muscular myosin (NMY-2::eGFP, green) can be observed after the first, second, and third round of divisions. At the two-cell stage, AB (left) has higher signal than P1 (dotted circle), and the P1 descendants EMS and P2 (dotted circle) have lower signal than the AB descendants (left and top). The top row of figures indicates the positions and names of the cells. At the eight-cell stage, the P2 descendants P3 (right, very dim, dotted circle) and C (not visible), as well as EMS descendant E (arrowhead) have lower signal than the AB descendants (ABal bottom-left, ABar top-left, and ABpr top-right). Bar indicates 5 μm (first image at the bottom). **(B)** Quantification of the cortical signal of NMY-2 and F-actin in the cells at the eight-cell stage. Data represent four embryos measured over time and are normalized for differences in intensity between dyes, embryos, background signal, imaging depth, the effect of bleaching due to imaging, and corrected for repeated measures (see section 2). F-actin and NMY-2 behave roughly similar, though NMY-2 differences are smaller. Both signals are asymmetrically distributed in the EMS daughters E and MS, with markedly lower signal in E (*p* < 0.005). Error bars represent standard error around the mean. **(C)** The relative F-actin signal changes abruptly during the EMS division. EMS, E, and MS cortex for four embryos are measured over time; signal is corrected for several confounders and repeated measures. **(D)** Heatmap representing the intensity of F-actin signal in the cortex during the EMS division to MS (anterior) and E (posterior). The heatmap represents a 2D spline fit to intensity data taken from image stacks with 5 s intervals from three embryos, corrected for systematic signal intensity differences between embryos. The furrow center (at the origin) is defined by the location of the membrane at the center of the cell at the moment of membrane closure. During cytokinesis the strong curvature of the membrane induces a temporary loss of signal from the focal planes (around t = 0). The contour lines indicated lines with identical intensity values. The top arrow highlights rapid signal loss on the posterior side of the cell. The lower left arrow points to the signal in MS, lower right the much lower signal in the E cell. **(E)** The cortex of EMS shows large differences in cortical flow between the anterior and posterior sides relative to the cytokinetic furrow. The data is based on manual tracking of cortical features of three embryos. Image stacks are 5 s apart.

Analyses and plots were made using R (version 3.6.3) and via the packages *gam, nlme, lme4*, and *emmeans*, unless indicated otherwise. For Figures 1B,C, the intensity signal was corrected in several steps. First, background signal (areas surrounding the cortices) was measured and subtracted from the cortical signal for every embryo, timepoint, and plane. Second, the signal *I* is corrected for systematic intensity differences between embryos were corrected by measuring the average intensity across the cells of the signal per embryo, and calculating a scaling factor *s*_*e*_ for every embryo *e*: $s_{e}=\frac{\frac{1}{mn}\overset{n}{\underset{i}{\sum }}\overset{m}{\underset{j}{\sum }}I_{i,j}}{\frac{1}{m}\overset{m}{\underset{j}{\sum }}I_{i,j}}$, with *n* the number of embryos, *m* the measurements per embryo. Measurements for an embryo are then multiplied by this value to yield corrected values $I_{c,e}^{*}$. Third, effects on signal due to time of imaging (bleaching) and depth of the imaging plane (signal degradation), were corrected by fitting a GAM regression model *f*(*t, z*) on the data for all embryos with a linear effect for bleaching (*t* time of imaging) and a smoothing spline to the log of the plane (*z*). The correction for an observation was calculated and scaled as $I_{c,e}^{+}=\frac{I_{c,e}^{*}-f\left(t,z\right)+\bar{I^{*}}}{\bar{I^{*}}}$. Finally, a model is fit on the corrected $I_{c,e}^{+}$ to estimate relative intensity values for individual cells using the *gls* function, taking into account time correlation between intensity measures using a first order autocorrelation structure (*corAR1* in *gls*). For Figure 1B, least square means and their standard errors are estimated on the model by *emmeans* with the Satterthwaite method and sampling to estimate variance components (mode *appx-satterthwaite*). For Figure 1C, a quadratic function is plotted which was fitted over time per cell using the *gls* function, again with a corAR1 correlation model. The plotted standard error over the fit is estimated based on the model's covariance matrix calculated by the *vcov* function.

When comparing volume and sphericity over time (**Figure 3**, Supplementary Figures 1, 2), mixed effect models were fitted with *lmer*, with random effects capturing embryo specific effects and evaluating temporal correlations. Volume ratios were modeled as described in the paper describing the segmentation methodology (Thiels et al., 2021). The model for sphericity of the E-cell using the *lmer-*package syntax is expressed as:


sphericity∽1+time+category+(1|embryo)

The fixed effects are given by a time component and a categorical variable “*category*” (wild-type or *dsh-2/mig-5* mutant), while the random effect accommodates the variation between embryos. This model was arrived at after model simplifications. A random effect per embryo on the time coefficient was considered, but was removed as it was not significant (χ^2^-test, *p* = 0.2).

The Figures 1D,E and the graphs in **Figure 4** where made with Python (version 3.7). Figure 1D was made by fitting a 2D tensor spline (package *pygam*, function *LinearGAM*) on the intensity data. Data was standardized per embryo (subtracting mean, division by standard deviation). The fitted spline was plotted using *contourf* from the package *matplotlib*.

Figure 1E was made based on manually tracking cortical features using the same custom tool as described for the PIV analysis above. The velocities of the features were next averaged and plotted using Python and the *quiver* function from package *matplotlib*.

**Figure 4B** is made by fitting a natural spline to normalized intensity data using Python and the package *statsmodels*.

For **Figure 4C**, the signal intensity was corrected by subtracting background (cytosolic) signal and scaled to the observed range of values across the contact area. The figure was made with the visualization package *seaborn* and the used test is the mixed two way anova test using Python and the *pingouin* package, with correction for repeated measures within embryos.

###  Analysis of Cellular Positioning

To follow cellular positioning over time, we imaged, tracked and analyzed histone labeled nuclei in embryos of the RW10029 as described before (Dzyubachyk et al., 2009; Krüger et al., 2015; Jelier et al., 2016), for both wildtype and RNAi treated embryos. To precisely establish differences in cellular positioning, a stringent alignment protocol was used. Time alignment was done by taking the last timepoint of the eight cell stage, which was the timepoint before division of either ABpl, ABpr, ABar, or ABal. Next, embryos were aligned in space by Procrustes analysis, which scales, centers and rotates the embryos to minimize the overall distance between nuclei. A Generalized Procrustes Analysis was performed to make a reference embryo for the wild-type embryos. Next all embryos were aligned by Procrustes Analysis to this reference. Finally, the distances across the different axes of the embryo (anterior-posterior, dorsal-ventral, left-right), were measured for every cell between all embryos and the reference embryo. The non-parametric two-sided Wilcoxon test was used to compare wild-type to the RNAi treated embryos.

**Figure 3D** was made with a custom tool (Java FX) to visualize and explore lineages, and shows a single RNAi embryo compared to the reference. **Figure 3E** is a scatter plot made in R and shows the positioning of ABpl in the aligned embryos relative to the reference along the anterior-posterior and dorsal-ventral axes.

## Results

To observe dynamical cortical behavior in the early embryo we used a strain (RJ012) expressing the F-actin binding peptide Lifeact tagged by the fluorophore mKate2 and non-muscular myosin (NMY-2) tagged with GFP, through modification of the endogenous *nmy-2* locus. As previously described (Reymann et al., 2016), we observed asymmetric levels of F-actin and NMY-2 in the cellular cortex during the zygotic division (Figure 1A), with P1 having lower levels for both markers. The lower level for both markers is maintained for both P1 descendants at the 4-cell stage, the endomesodermal precursor cell EMS and P2 (Figure 1A, Supplementary Movie 1), though the effect is more pronounced for the F-actin marker. The pattern propagates into the eight-cell stage, where three of the four P1 descendants have lower signal. However, the MS cell breaks with the pattern and has a relatively high level for both markers (Figure 1B). By monitoring the F-actin signal in the cortex of EMS and its daughters over time (Figure 1C), it becomes clear that the differences between E and MS arise during the division. To better characterize the phenomenon, we proceeded to follow the intensity of cortical F-actin during the EMS division with high temporal and spatial resolution (Figure 1D). Starting about 5 min prior to completion of cytokinesis, a gradient of F-actin signal arises across the EMS cell, with a rapid signal decrease on the posterior side and signal peaking just anterior to the cytokinesis cleft. After cytokinesis completes, the cortex of MS maintains a higher signal compared to E. We also observed highly dynamic cortical flow changes during cell division. In Figure 1E we represent the cortical flow based on manually tracked features of F-actin visualized by Lifeact. Nine hundred and sixty cortical features were traced, totaling 2,596 datapoints across three embryos. Until 4.5 min before completion of cytokinesis, EMS has a homogeneous flow to the dorsal side. Then the flow redirects to a posterior to anterior flow and a slight asymmetry in speed arises between the anterior and posterior halves of the cell (270–180 s before cytokinesis). Finally, a marked asymmetry arises between the anterior and posterior of the cell (from −180 s till completion of cytokinesis). The flow speeds up in the posterior, whereas it comes to a near standstill in the anterior. Further, we observed differences in duration at which distinct features (F-actin fragments) are visible in the cortex, with a much shorter feature life time for the posterior side of the EMS cell (E side). During the last 3 min before cytokinesis completes, features are visible during ~25 s for the anterior side, vs. ~13 s for the posterior side (H0 of no difference rejected at *p* < <0.001, oneway ANOVA), which indicates a more dynamic F-actin network in the posterior cortex.

**Figure 2:**
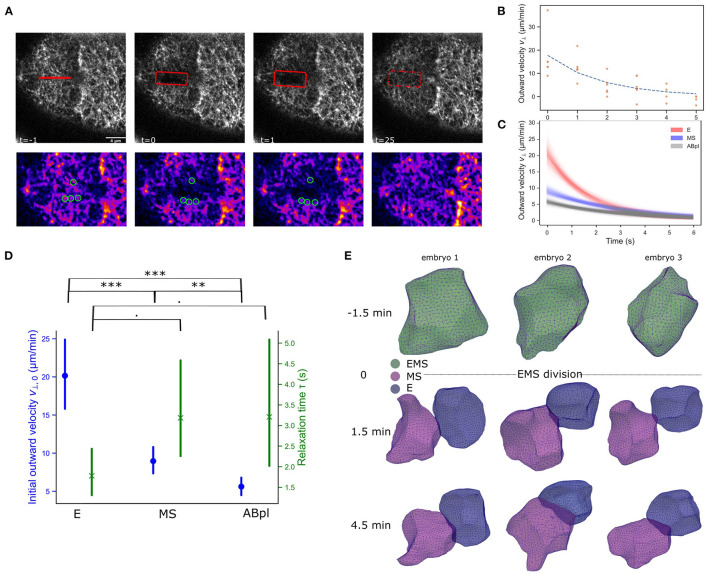
The differences in the NMY-2 and F-actin markers across E and MS coincide with cortical tension differences and cell shape changes. **(A)** Illustration of cortical ablation experiments on an E cell. A 4 μm cut is made by a pulsed 355 nm laser (UGA-42 Caliburn). In the sequence the opening of the cut, highlighted with the rectangle can be observed, followed by repair of the cortical disruption. The second row shows the manual PIV measurement for this experiment. The image is processed (blurring and background subtraction), and a color LUT is used to better see intensity contrasts. Cortical features are marked with green circles. A modest recoil is noted, and after ±25 s the cut is repaired. Scale bar is 4 νm. **(B)** Output of the PIV analysis. An example of outward velocities for an E cell are shown with an exponential trend fit. The movement is quickly reduced to low levels and the maximal gap opening is achieved after 3–5 s. **(C)** Exponential fit for the ablations of E, MS, and ABpl cells, with confidence interval. The analysis is based on 4, 7, and 7 experiments respectively. The E and MS cells show different initial velocities, which indicates E has larger initial tension than MS. For reference, also the ABpl cell was ablated, which has still higher F-actin/NMY-2 than MS (cf. Figure 1B), and this cells shows very little cortical tension. **(D)** Statistical analysis of the cortical ablation experiment, showing both the initial outward velocity estimate and the relaxation time. Bars represent 95% confidence level and test results are based on a permutation test (weighted bootstrap, see section 2). · *p* < 0.1, **p < 0.01, ***p < 0.001. **(E)** E and MS take different cellular shapes after division. Cells were reconstructed from images of embryos with membranes tagged by a membrane binding domain fused to GFP.

Cortical flows are associated with anisotropies in cortical tension across the cell (Mayer et al., 2010). However, the observed differences in F-actin and NMY-2 in the daughter cells also point to a durable restructuring of the cellular cortex after division. We should interpret Lifeact intensity results with caution as we can not exclude that the Lifeact peptide is an imperfect indicator of actual quantities of F-actin in the descending cells (Hirani et al., 2019). Perhaps the asymmetric inheritance of bound peptide during the division severely reduces the concentration in the E cell, or maybe the Lifeact peptide has relatively slow F-actin binding dynamics in the apparently highly dynamic cortex of the E cell. Nonetheless, the NMY-2 marker is a fusion to the endogenous gene, which can be expected to be accurate, and it shows the same trend although with smaller changes. To test whether the observed changes in marker abundance after division of EMS translate into differences in mechanical properties of the cortex of E and MS. We performed ablation experiments of the cortex of these cells at the eight-cell stage, and followed the opening and closing of the ablation cut over time (Figure 2A). Typically, the cortex's response to ablation is modeled by assuming that it behaves as a 2D active viscoelastic gel (Saha et al., 2016). A typical cortical ablation analysis aims to quantify the evolving opening speed of the cut, with an expected exponential decay (Figure 2B). The initial recoil speed orthogonal to the cortical cut upon ablation is proportional to the stress across the cut (Mayer et al., 2010; Saha et al., 2016). Further, the speed decay over time is related to the stiffness of the cortex, with a lower relaxation time indicating a stiffer cortex (Mayer et al., 2010). As shown in Figures 2C–E has a higher initial outwards velocity than MS, indicating a considerably higher cortical tension, approximately twice that of MS. The ABpl cell, which is F-actin rich and is a relatively spread out cell at this stage, has an even lower velocity and cortical tension. The E cell also appears to have a stiffer cortex than MS and ABpl as manifested through the lower relaxation time, though the effect only has a marginal significance level (*p* < 0.1, Figure 2D). To obtain more information on the nature of these differences we also considered the shape of the cells. The motivation is that high overall cortical tension makes cells more rigid and spherical, similar to when cells round up prior to mitosis by increasing cortical stiffness and tension (Stewart et al., 2011), whereas lower cortical tension allows more flexible and irregular shapes. We employed a novel cellular segmentation pipeline to retrieve the shapes of the cells from confocal microscopy images of embryos with fluorescently membranes (strain RJ013) (Thiels et al., 2021). Over time, E retains a mostly spherical shape, whereas MS develops a more irregular shape, and in some embryos forms a lamellipodium structure 4.5 min after EMS division (Figure 2E, MS lamellipodium in embryos 1 and 2, see Supplementary Figure 1 for sphericity measures). Both observations, combined with the assumption that the friction experienced by the cortex is relatively stable in the short time frame, point to a higher cortical tension in E.

**Figure 3:**
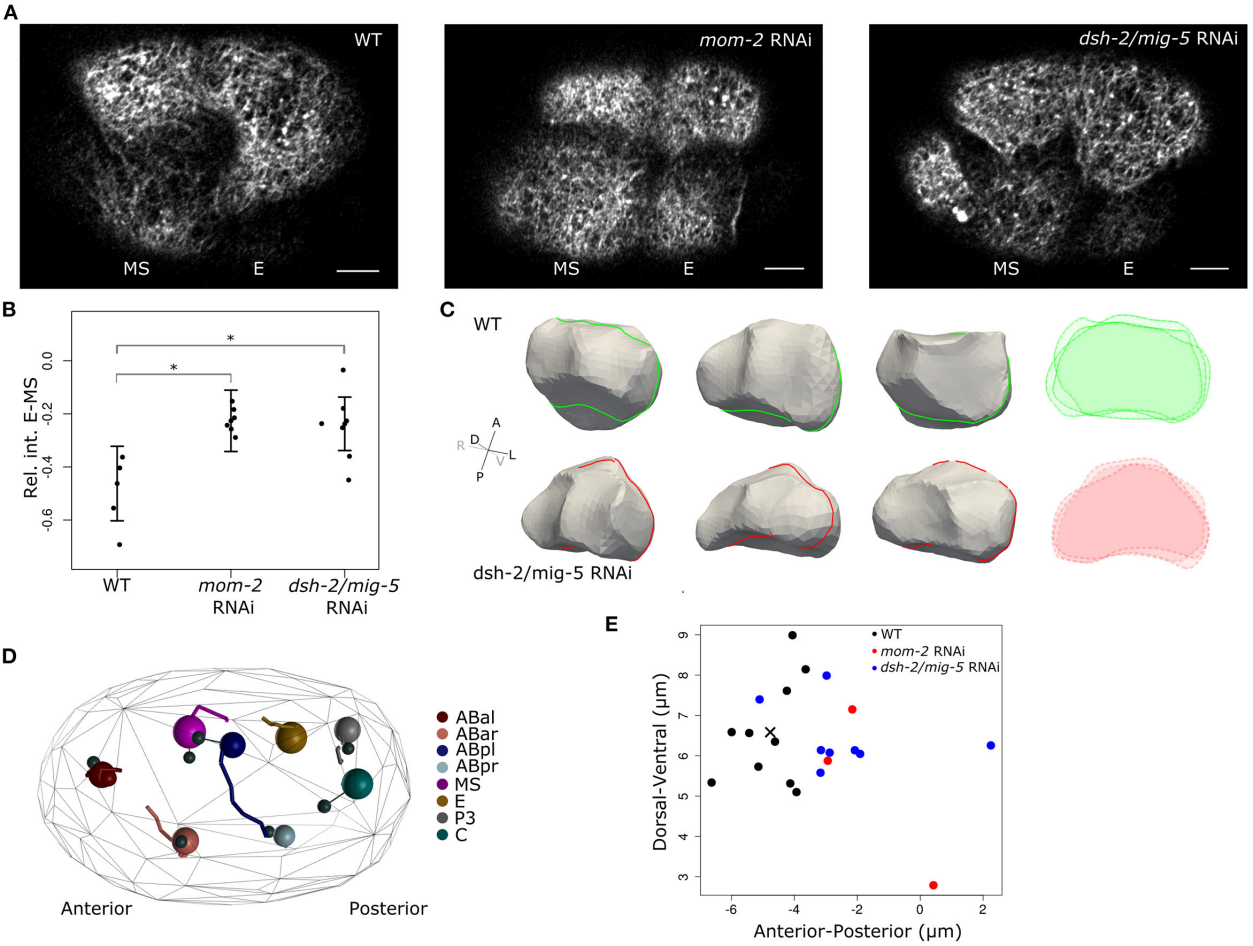
Wnt signaling drives asymmetric cortical behavior during and after EMS division. **(A)** Cortical images showing effect on F-actin distribution when Wnt signaling is disrupted. F-actin shows nearly symmetric distribution between the E and MS daughters when either the Wnt ligand (*mom-2*) or the disheveled proteins (*dsh-2, mig-5*) are knocked down. Images are taken 3–5 min after completion of cytokinesis. Scale bars indicate 5 μm. **(B)** Quantification of the effect of disruption of Wnt signaling on F-actin asymmetry between E and MS. Cortical intensity is measured and corrected as before, the shown values are the difference in intensity between E and MS (E-MS). Error bars represent the standard error and are centered around the mean. **(C)** Shape reconstruction of the E cell in both wildtype and *dsh-2/mig-5* RNAi embryos 3' after EMS division, with overlaid intersections shown in the right panel. At the shown moment, the perturbed embryos have more irregularly shaped E cells, with in some embryos a marked indentation at the posterior side by P2 as it is going through division. **(D)** Disrupting Wnt signaling induces a minor positional phenotype at the eight-cell stage. Systematic analysis of cellular positioning by lineaging 8 embryos treated with *dsh-2/mig-5* RNAi showed a slightly more posterior positioning of the ABpl cell. The large spheres represent the cells in a perturbed embryo. The positioning in the reference embryos is shown by connecting the cells to small black spheres that represent the cells' average position in reference embryos. The movements of the cells are shown by traces in the same color. **(E)** Position of ABpl in wild type and RNAi embryos. ABpl is consistently positioned more to the posterior when the Wnt signal is disrupted by RNAi for either the Wnt ligand (*mom-2*) or the Dsh proteins (*dsh-2/mig-5*) (cross indicates the average WT position). The cross marks the average position of ABpl in the reference embryos. *indicates significance at the 0.05 level

As the EMS cell is polarized by the Wnt and MES-1 signaling pathways, which induces the E fate in the posterior daughter born next to P2 (Goldstein, 1992; Bei et al., 2002), we asked if the Wnt signaling induces the cortical flows and reorganization of the cortex. Figure 3A shows the results of Wnt pathway knockdown by RNAi for the Wnt ligand *mom-2* and RNAi for *dsh-2* and *mig-5*, two genes coding for the disheveled proteins active during early embryogenesis. The disheveled proteins relay the signal coming from the Frizzled receptors upon binding the Wnt ligand (e.g., MOM-2) to distinct cellular responses. Both RNAi experiments resulted in the near equalization of F-actin signal during, and after the EMS division (Figure 3B) with high penetrance (*mom-2* RNAi: 7/8; *dsh-2/mig-5* RNAi: 24/34). Though both RNAi experiments perturb Wnt signaling and equalize the division with respect to F-actin, we found they varied in the extent that they affect the fate induction of the E cell. RNAi of *mom-2* invariantly inhibited E fate induction, perturbing the ingression of the cells during gastrulation. RNAi of the *dsh-2/mig-5* did not have the same effect, with gastrulation of Ea and Ep proceeding normally, including the fate-specific delayed division of these cells (Sulston et al., 1983). This indicates therefore that the F-actin distribution in EMS is not essential for the fate induction of the E cell.

To evaluate whether Wnt signaling has an effect on the cell shape, we segmented cells following EMS division in five *dsh-2/mig-5* RNAi embryos, and compared to seven wildtype embryos. The perturbation does not remove the volume asymmetry in the EMS division (Supplementary Figure 2A), but instead increases it slightly. However the perturbation does make the E-cell shape more irregular, and decreases sphericity (*p* < 0.05, mixed effects model with χ^2^-test, Figure 3C, Supplementary Figures 2B, 3), which points to a drop in cortical tension.

We next asked if there is a direct contribution of the asymmetry in EMS to the cellular movements occurring around, and after EMS division. We therefore precisely tracked cellular movements, division timings and division angles during the four and eight-cell stage by lineaging wildtype and RNAi treated embryos (Bao et al., 2006; Krüger et al., 2015; Jelier et al., 2016). By comparing eight RNAi to ten wildtype embryos, we found very limited phenotypes in these early stages, but we did observe a modest mispositioning of the ABpl and ABar cell, with the former ending up positioned posterior to their normal location at the end of the eight-cell stage (Figure 3C). The ABpl cell moves a long way across the embryo during the seven to eight-cell stage as part of the formation of a cellular arrangement that is important for left-right asymmetry in the embryo (Pohl and Bao, 2010). ABpl forms a lamellipodium into the cytokinesis groove of the dividing EMS cell preceding this movement, and specifically associates with MS. In our hands, the formation of the lamellipodium or the size of the movement of the ABpl cell is not reduced due to the RNAi perturbation of Wnt signaling. However, ABpl is modestly and consistently misdirected to the posterior side for both *mom-2* and *dsh-2/mig-5* RNAi treatments (Figures 3D,E, *p* < 0.001, Wilcoxon rank sum test).

**Figure 4:**
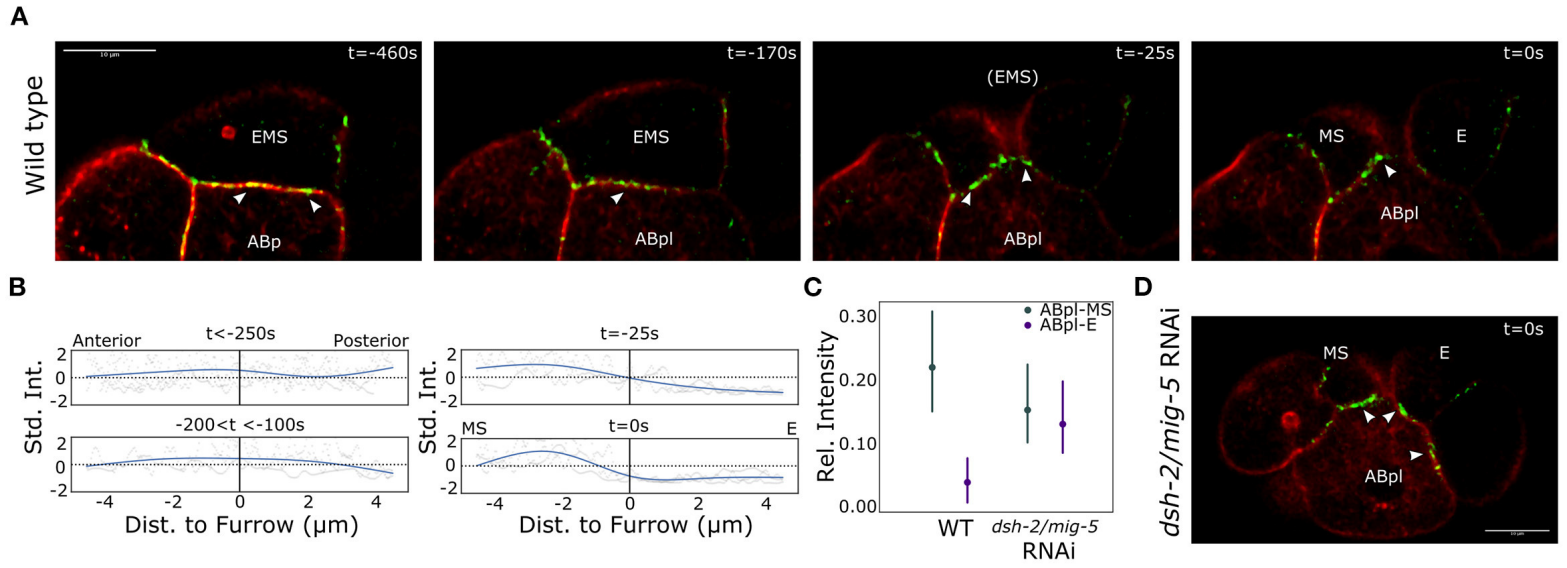
Asymmetric E-cadherin distribution coincides with the asymmetric cortical dynamics in EMS. **(A)** Distribution of the E-cadherin HMR-1 across the interface between EMS and ABp/ABpl. Note that ABpl forms a lamellipodium that precedes the ABpl movement following EMS division. Life act marking F-actin in red, the E-cadherin HMR-1 in green. Arrow heads indicate HMR-1 foci as mentioned in the text. Scale bar indicates 10 μm. **(B)** Quantification of the HMR-1 distribution over the ABpl/EMS interface. A natural spline is fit on the normalized data from three wildtype embryos. **(C)** The HMR-1 asymmetry is controlled by the Wnt signal. The RNAi effect on the distribution across the two cells is significant (*p* < 0.005) in a mixed 2-way ANOVA test. Data from seven wildtype embryos and three *dsh-2/mig-5* RNAi treated embryos. The signal is corrected for background, normalized for differences in signal range, and the model includes a random effect to capture embryo specific effects. Bars indicate 95% confidence interval. **(D)** Illustration of the HMR-1 signal in E and MS after completion of cytokinesis upon RNAi treatment for the disheveled genes *dsh-2/mig-5*. Colors are as for **(A)**.

As the ABpl cell moves over MS and E, it most likely exerts a force on either or both of these cells. To exert such a force, cell adhesions are required. As the E-cadherin HMR-1 is known to play a role in cell adhesion in early embryogenesis, we decided to image the localization of HMR-1 during EMS division. For this purpose we used a strain expressing the endogenous HMR-1 fused to GFP as well as the tagged F-actin marker Lifeact::mKate-2 (strain RJ001). We observed that HMR-1 distribution across the EMS-ABp(l) contact is highly dynamic (Figure 4A). Initially the distribution is homogeneous across the cell contact. As the EMS division approaches, the HMR-1 distribution becomes polarized and accumulates more on the anterior side of the interface. Just before cytokinesis of EMS completes, the HMR-1 signal abruptly disappears along the future E-ABpl interface. In Figure 4B, the distribution of HMR-1 across the ABpl/EMS interface is characterized over three embryos. We next asked if this distribution also depends on Wnt signaling. Figures 4C,D show the effect of *dsh-2/mig-5* RNAi on the distribution of HMR-1. The asymmetry in the distribution across the ABpl-EMS interface is completely removed and thus depends on the polarization driven by Wnt signaling. The phenotype was seen in six out of seven observed *dsh-2/mig-5* RNAi treated embryos. Concluding, Wnt signaling drives asymmetric distribution of E-cadherin across E and MS, which plays a role in ABpl specifically interacting with MS.

## Discussion

During EMS division, the cellular cortex undergoes active reorganization prior to cytokinesis, with cortical flows and a shift in apparent F-actin and NMY-2 distribution. This behavior appears similar in nature to the cortical flow during the pseudo-cleavage furrow phase of first zygotic division where the cell polarity of the cell is established (Reymann et al., 2016). Like the zygotic division, the EMS division is asymmetric in the fate and volume of the daughter cells, and the spindle orientation and spindle location are tightly regulated. The way polarization is induced differs, with the zygote being polarized by the centrioles donated by the sperm, whereas the EMS cell is polarized by signaling from the posterior P2 cell (reviewed in Rose and Gönczy, 2014). Also, during the zygotic division, the cortical flow is associated with the displacement of PAR proteins, the subsequent establishment of asymmetric AP cortical domains and the unequal segregation of cell fate determinants (Rose and Gönczy, 2014). This system plays no similar role in the EMS cell given that the PAR proteins are not distributed along the EMS polarization axis (Nance and Priess, 2002). Instead the polarization and fate change are induced by Wnt and Src signaling from P2. The signaling induces an asymmetrical distribution of several Wnt pathway proteins in the so-called Wnt/β-Catenin asymmetry pathway, specifically APC (APR-1) and the β-Catenin WRM-1 to the anterior (MS) side, and Frizzled and Disheveled homologs remain to the posterior (E) side (reviewed in Sawa and Korswagen, 2013; Lam and Phillips, 2017). Interestingly, the anterior polarizing movement of APR-1 coincides with the cortical flow (Heppert et al., 2018), and transport of this protein may be one particular role of the flow. We show here that perturbation of the Wnt ligand (*mom-2* RNAi) or the disheveled proteins (*dsh-2/mig-5* RNAi) nearly abolishes the cortical flow and NMY-2 and F-actin distribution asymmetries. A similar observation on the cortical asymmetry has been reported following RNAi for Wnt signaling genes for the zygotic division (Naganathan et al., 2014). The disheveled proteins can modulate the F-actin network indirectly, for example by activating the small GTPases RHO and RAC in the context of the planar-cell-polarity cascade, which then activate downstream targets to modulate the actin cytoskeleton, or through interactions with formins that can induce actin nucleation and elongation of actin fibers (reviewed in Wallingford and Habas, 2005). The cortical asymmetry is not essential for the fate induction in EMS as the knockdown of the disheveled proteins left E-fate associated properties, such as delayed division of Ea/p and gastrulation time, in place. This matches earlier reports of low penetrance (~3%) for *dsh-2/mig-5* RNAi causing defects in the endoderm fate induction (Liu et al., 2010). This limited effect has been attributed to simultaneous MES-1 signaling from P2, which provides a redundant pathway to induce the E-fate (Bei et al., 2002).

We verified by ablation that the properties of the cortex in the daughter cells remain different after the division, with higher cortical tension in the E cell. These changes are supported by observations of the cell shape of E and MS, where the former remains spherical after division and the latter rapidly changes shape and repositions. The higher cortical tension in the E cell is reduced upon *dsh-2/mig-5* RNAi, with the E cell showing more deformation upon pressures from neighboring cells. It is interesting to consider that the differences in the cortical behavior and physical properties between E and MS play a role in the robust cellular positioning in the early embryo, with the lower effective tension of MS facilitating the changes in shape and movement of the cell.

We found that perturbing the Wnt signaling with RNAi induces only a modest positional phenotype at the eight-cell stage, with a small displacement of ABpl to the posterior, while maintaining the overall movement of the cell. This led us to postulate that the way ABpl exerts forces on the E and MS cells has changed, for instance by having a stronger adhesion to the E-cell. By following E-cadherin on the cortex of EMS and descendants we indeed observed that cytokinesis is associated with a marked displacement of E-cadherin toward the anterior descendant MS. In the wildtype embryo, ABpl moves over and associates with MS, with foci of the E-cadherin HMR-1 at the interface. Whereas upon perturbation of the Wnt signaling by *dsh-2/mig-5* RNAi, the HMR-1 distribution shifted to the posterior E cell. The cortical dynamics and flow are likely directly associated with the distribution of E-cadherin between E and MS, as cortical flows can transport E-cadherin. An example is the basal-to-apical flow of cadherin at cell junctions (Kametani and Takeichi, 2007), where E-cadherin latches on to F-actin through α-catenin, which itself binds to E-cadherin by mediation of β-catenin. Further, local contractility can lead to accumulation of cortical F-actin and aggregation of cortical proteins (Munjal et al., 2015). During the EMS division the cortical flow could transport HMR-1 to the anterior side of the cytokinetic furrow, and the transient F-actin accumulation on the anterior may play a role in aggregating HMR-1 at this location. HMR-1 can also modify the cortical contractility and flow, as in the *C. elegans* zygote, where HMR-1 has been reported to slow down cortical flows by drag and negatively regulate RHO-1 activity, a GTPase associated with recruitment and activation of myosin II (Padmanabhan et al., 2017). The accumulation of HMR-1 to the anterior side of the cytokinesis cleft may therefore also play a role in slowing down the cortical flow.

It is interesting that a lower apparent amount of F-actin and NMY-2 in E vs. MS appears to be associated with a higher cortical tension in the former. This is in contrast to the zygotic division, a higher tension is generated by the anterior part of the cell, which has higher F-actin and NMY-2 signal than the posterior side (Mayer et al., 2010). However, there are many facets that modulate the activity of the cortex, and the mechanisms of the observed difference remain to be elucidated. It is possible that the active ATP-driven force generation is upregulated in E, e.g., by local NMY-2 phosphorylation (Wei et al., 2020), which could drive up the tension, irrespective of the lower density. The difference may also be caused by differences in crosslinking in the cortex, as crosslinking is a key regulator of actomyosin contractility (Inoue et al., 2011; Krueger et al., 2019). Further, cortical tension is hypothesized to be maximal for an intermediate level of connectivity in the actomyosin network (Chugh et al., 2017; Ding et al., 2017). When connectivity is too high or too low, the generated tension in the network will be low (Ennomani et al., 2016), and a high level of connectivity has been suggested to make the network incapable of transmitting tensions over larger distances (Ennomani et al., 2016). This line of reasoning aligns with the dense F-actin networks we observe in MS and the AB descendants.

We conclude that during EMS division a cortical flow arises, as well as a dynamic transition in the cortical contractile network and E-cadherin distribution. The abrupt change in cortical dynamics, during and after EMS division, is driven by Wnt signaling, and the observed cell state transition is associated with changes in cortical tensions, cell shape and actomyosin organization of the descendant cells. The mechanisms underlying such transitions are an active research area, and further study of this asymmetric division could unveil new insight into the determinants of actin network architecture.

## Data Availability Statement

The original contributions presented in the study are included in the article and Supplementary Material, further inquiries can be directed to the corresponding author.

## Author Contributions

FC made the initial observation and performed the experiments. MV performed part of the ablation experiments. WT performed the cell shape analyses. RJ supervised, performed image analyses, lineage tracking and the statistical analyses, and wrote the paper. All authors contributed to the article and approved the submitted version.

## Conflict of Interest

The authors declare that the research was conducted in the absence of any commercial or financial relationships that could be construed as a potential conflict of interest.

## Publisher's Note

All claims expressed in this article are solely those of the authors and do not necessarily represent those of their affiliated organizations, or those of the publisher, the editors and the reviewers. Any product that may be evaluated in this article, or claim that may be made by its manufacturer, is not guaranteed or endorsed by the publisher.
