# Supplementary material for: Wnt Signaling Induces Asymmetric Dynamics in the Actomyosin Cortex of the C. elegans Endomesodermal Precursor Cell
Source: Front Cell Dev Biol. 2021 Sep 17;9:702741. doi: 10.3389/fcell.2021.702741 (PMC8484649; doi:10.3389/fcell.2021.702741)
Supplement: Supplementary file 1 [file Data_Sheet_2.PDF]

Supplementary Figures

Movie 1: The movie shows a timelapse of a developing embryo with marked F-actin (Lifeact, red) and non-muscular myosin (NMY-2::eGFP, green) from after the first till after the third round of divisions. At the two-cell stage, AB (left) has higher signal than P1 (right cell), and the P1 descendants EMS and P2 (see Figure 1A for help with cellular identification) have lower signal than the AB descendants (left and top). At the eight-cell stage, the P2 descendants P3 (right, very dim, dotted circle) and C (not visible), as well as EMS descendant E (arrowhead) have lower signal than the AB descendants (ABal bottom-left, ABar top-left and ABpr top-right). Bar indicates 10 μm.

Figure 1: Shape analyses of the E and MS cells. The sphericity of the cells is markedly different. Both cells take more irregular shapes after division, but E maintains a considerably more spherical cell than MS. The analysis is based on data from 7 embryos.

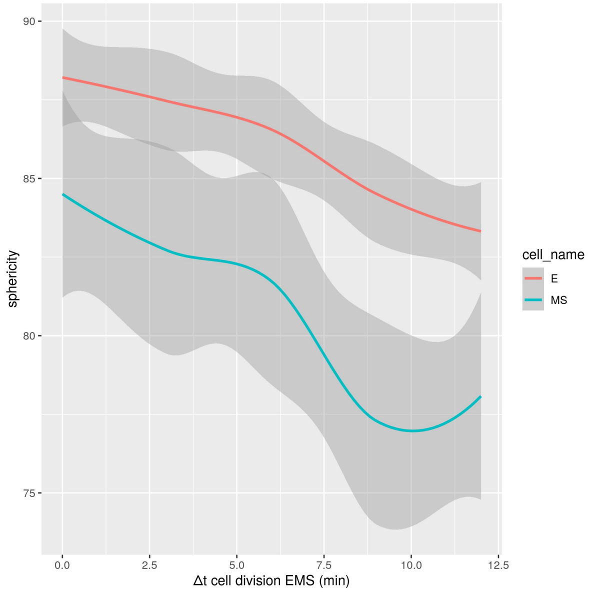

Figure 2: Shape analyses of cells after EMS division. A. Asymmetry in volume following division is maintained for E/MS following *dsh-2/mig-5* RNAi; 95% confidence interval shown. There is evidence for a slight increase in the volume asymmetry, while in P2 division the volume is not affected. B. Sphericity is lower following the *dsh-2/mig-5* RNAi for the E cell. The effect is clear at 3' after EMS division, when the dividing P2 cell pushes into the E cell.

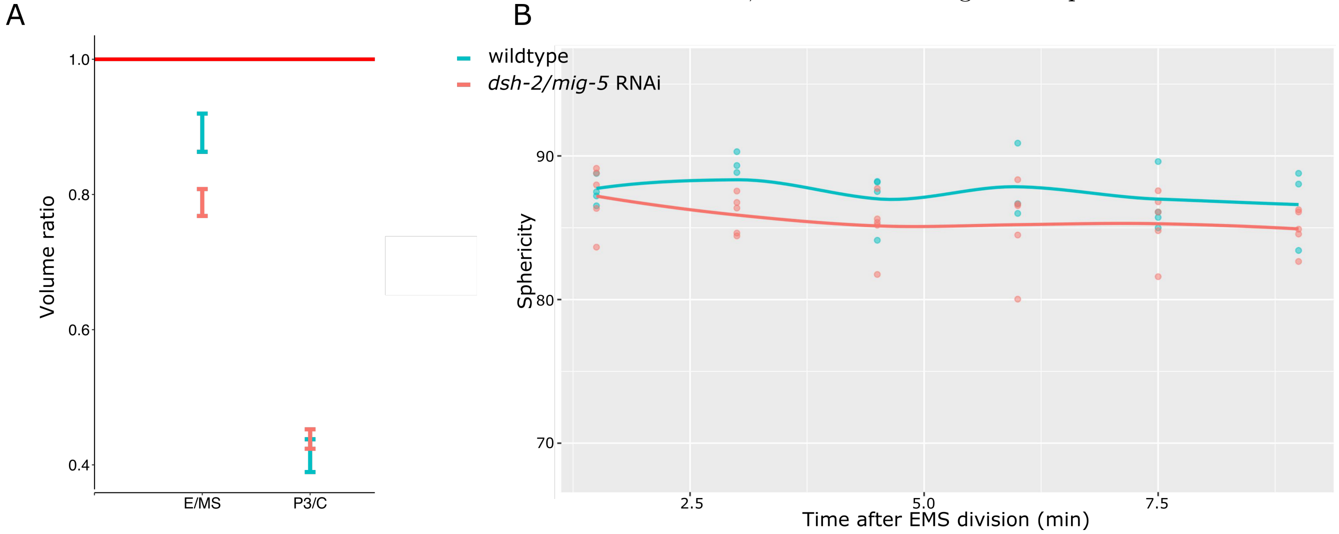

Figure 3: Examples of the differences in E-cell shape between wild-type and *dsh-2/mig-5* mutants, three minutes after EMS-division. For context, the MS cell is also shown. The E-cell of the *dsh-2/mig-5* mutant, on average, is more flattened compared to wild-type.

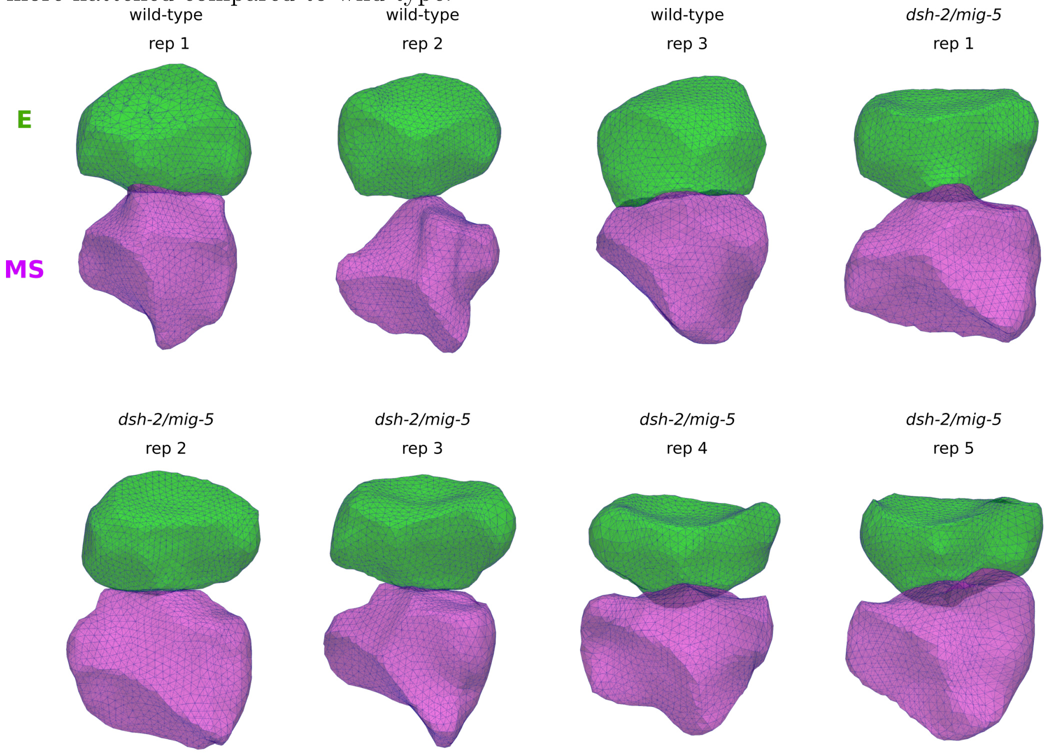

Table 1: Statistics on PIV manually annotated markers

Cell	# embryos	# markers
E	4	105
MS	7	153
ABpl	7	168

Table 2: Values underlying statistical analyses for the cortical ablation contrasts (Figure 2D) for initial outward velocity $v_{\perp,0}$ and relaxation time τ.

cell pair	parameter	observed difference	p-value	significance
E/MS	$v_{\perp,0}$	11.2	< 0.001	***
E/MS	τ	1.41	0.051	.
E/ABpl	$v_{\perp,0}$	14.5	< 0.001	***
E/ABpl	τ	1.43	0.065	.
MS/ABpl	$v_{\perp,0}$	3.3	0.007	**
MS/ABpl	τ	0.02	0.98	
